# Renal Sinus Pathologies Depicted by CT Imaging: A Pictorial Review

**DOI:** 10.7759/cureus.57087

**Published:** 2024-03-27

**Authors:** Vlad-Octavian Bolocan, Georgian-Florentin Diaconu, Mihaela Secareanu, Loredana Sabina Cornelia Manolescu, Viorel Jinga, Maria-Glencora Costache, Gelu Adrian Popa, Cosmin Medar

**Affiliations:** 1 Department of Clinical Laboratory of Radiology and Medical Imaging, Clinical Hospital “Prof. Dr. Theodor Burghele”, Bucharest, ROU; 2 Department of Fundamental Sciences, Faculty of Midwifery and Nursing, University of Medicine and Pharmacy “Carol Davila”, Bucharest, ROU; 3 Department of Urology, Clinical Hospital “Prof. Dr. Theodor Burghele”, Bucharest, ROU; 4 Department of Urology, Faculty of Medicine, University of Medicine and Pharmacy “Carol Davila”, Bucharest, ROU; 5 Medical Sciences Section, Academy of Romanian Scientists, Bucharest, ROU; 6 Department of Radiology and Medical Imaging, Clinical Hospital of Emergency “Sf Ioan”, Bucharest, ROU

**Keywords:** metal toxicity, urolithiasis, tumours, imaging, renal sinus pathologies

## Abstract

Diverse conditions comprise the spectrum of renal sinus pathologies, which have diagnostic and therapeutic implications for patients. Using CT imaging as a lens, this exhaustive review examines the representation of these pathologies. The article begins with a concise synopsis of renal anatomy and the specialized CT methodologies utilized to achieve excellent visualization. Transformational cell carcinoma, leiomyosarcoma, renal cell carcinoma, multilocular nephroma, and lymphoma are among the tumoral origins of the renal sinus pathologies that are investigated. Further, vascular pathologies including fistulas, hematomas, and aneurysms are included in the discourse, along with parapelvic and peripelvic cysts, and lipomatosis. In addition to urolithiasis and encrusted uretero-pyelitis, the review examines the consequences of metal toxicity and non-neoplastic conditions. With a focus on critical CT imaging findings that aid in the provision of an accurate diagnosis, every pathology is meticulously examined. With the intention of improving clinical decision-making and patient care, this article intends to function as a valuable resource for radiologists, clinicians, and researchers who are engaged in the interpretation and comprehension of renal sinus pathologies.

## Introduction and background

Anatomy

The renal sinus is a spacious central cavity that arises from the expansion of the perinephric space into a profound recess located at the core of the kidney. It is enveloped by the renal tissue on the lateral aspects. The renal sinus houses the primary branches of the renal artery and vein, as well as the major and minor calyces of the collecting system. It also contains fat, lymphatic veins, nerve fibers of the autonomic nervous system, as well as various quantities of fibrous tissue. Consequently, the different elements of the renal sinus might give birth to distinct clinical diseases. Moreover, the renal sinus can also be indirectly affected by clinical diseases that include the surrounding renal tissue and neighboring retroperitoneal regions [1-3].

Technique

The utilization of CT has evolved into an essential procedure for diagnosing renal sinus pathologies. A CT scan offers a thorough assessment of the renal sinus as well as its adjacent anatomical components, such as the collecting system, medulla, and cortex. The examination may be conducted with or without IV contrast; the selection of the contrast agent and the scheduling of the scan are determined by the particular pathology under investigation. CT scans are highly advantageous in two situations: identifying calcifications (e.g., urolithiasis) and evaluating the renal sinus in instances of heavy metal poisoning (e.g., extensive streaking artifacts caused by metal objects in the body).

In addition to the detection of calcifications, CT is also useful for the evaluation of renal sinus hematomas and urinomas. Hematomas appear as amorphous high-density substances on CT, highly suggestive of fresh blood. Urinomas, on the other hand, are readily demonstrated in contrast-enhanced studies on the excretory phase, due to direct contrast extravasation from the urinary tract. In cases of pyelonephritis, CT can demonstrate the presence of conglomerate inflammatory cells, leading to a pseudotumor within the renal sinus. The presence of encrusted uretero-pyelitis can also be suggested by the specific aspect of calcifications on CT scans. With the combination of these imaging features, CT provides a comprehensive evaluation of renal sinus pathologies, enabling prompt and accurate diagnosis and treatment.

CT is becoming a useful tool for diagnosing and treating renal sinus tumors. CT enables the evaluation of the dimensions, position, and correlation of the abnormality with nearby structures, as well as the identification of any potential spread into neighboring tissues or organs. Giving contrast material through an IV makes the lesion and the tissues around it clearer, making it easier to tell the difference between it and other structures and giving important information for getting ready for surgery.

Furthermore, CT can assist in distinguishing between harmless and cancerous growths, as well as offer insights into the severity of the cancer and its impact on the renal sinus vasculature. It is crucial to consider this specifically for the staging and therapy of renal cell carcinomas, which are the predominant form of renal malignancy and frequently arise in the renal sinus. A CT exam allows for the evaluation of tumor vascularity, necrosis, and hemorrhage, as well as provides crucial prognostic data.

Lesions involving the renal sinus can be classified as either non-neoplastic or neoplastic, similar to other anatomical areas. Tumors that affect the renal sinus can be classified into several categories based on their site of origin. These include tumors originating from the renal pelvis, tumors that spread from the renal parenchyma into the renal sinus, primary tumors of mesenchymal origin, and retroperitoneal tumors that ultimately impact the renal sinus. Non-tumorous conditions like lipomatosis, vascular lesions, cysts, and fluid collections, which can be either inflammatory or non-inflammatory, can affect the renal sinus [4].

## Review

Tumors

Various benign and malignant neoplasms can affect the renal sinus, either directly from it or via the surrounding cortex or retroperitoneum. The presence of fat in the renal sinus is useful in detecting tiny cancers. Observing the fat in the renal sinus can help identify the presence of a tumor and determine its stage. The presence or absence of fat can help diagnose and manage renal sinus neoplasms [1,3,5]. Only around 5% of urinary tract neoplasms are caused by malignant tumors in the renal pelvis.

Transitional cell carcinomas (TCCs)

TCCs account for 95% of all uroepithelial tumors in the renal pelvis. The remaining 5% consists of squamous cell carcinoma (the majority) and adenocarcinoma. TCCs can have one of two shapes: papillary (more than 85% of tumors) or non-papillary (tumors that are sessile or nodular, high-grade, and invade beyond the mucosa early). Papillary tumors have frond-like projections and are low-grade, with invasion beyond the mucosa happening later. Patients often appear with microscopic or macroscopic hematuria. If the tumor is in the pelviureteric junction, hydronephrosis symptoms, like pain in the flank, may be the first sign. Clots related to the condition may cause symptoms like those of renal calculi. Some people seek medical assistance only after their metastatic disease produces symptoms, such as constitutional symptoms or symptoms caused by a specific metastatic deposit [6].

**Figure 1:**
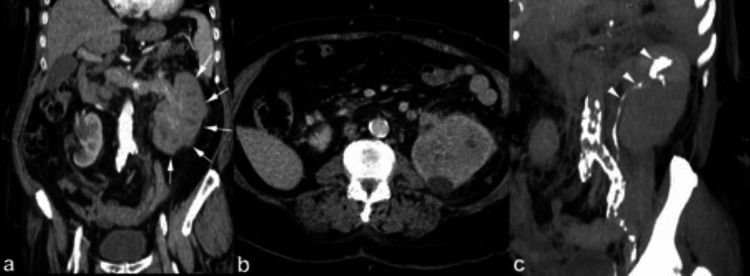
The contrast-enhanced CT (CECT) images during the arterial (a), nephrographic (b), and urographic (c) phases demonstrate the faceless kidney, which appears enlarged and displays a high density of soft tissue. Furthermore, the enhancement of the renal vein is modest due to tumoral invasion accompanied by indications of thrombosis. Large infiltrating transitional cell carcinomas (TCCs), as opposed to renal cell carcinomas (RCC), do not alter the kidney's inherent contour. Additionally, obliteration and amputation of the pyelocaliceal system are noted, which serve as indications of substantial pathological alterations occurring within the renal structure. This figure is a republished image, permission was obtained from the original publisher, https://epos.myesr.org/poster/esr/ecr2018/C-2802

TCCs (Figure 1) typically have soft tissue density (8-30 Hounsfield units (HU)) and modest enhancement, which is less pronounced than renal parenchyma or renal cell carcinoma. These tumors are typically located in the renal pelvis rather than the renal parenchyma and can range in size from minor filling deficiencies to massive masses that obliterate the renal sinus fat. Large infiltrating transitional cell carcinomas, as opposed to renal cell carcinomas, have normal renal morphology. Larger tumors may contain zones of necrosis. A small soft tissue mass should be sought in situations of minor tumors near the pelviureteric junction, which result in hydronephrosis. The calyces are usually dilated, and the renal pelvis wall can be thickened. Small calcifications may occasionally appear on the surface of papillary projections [7,8].

**Figure 2:**
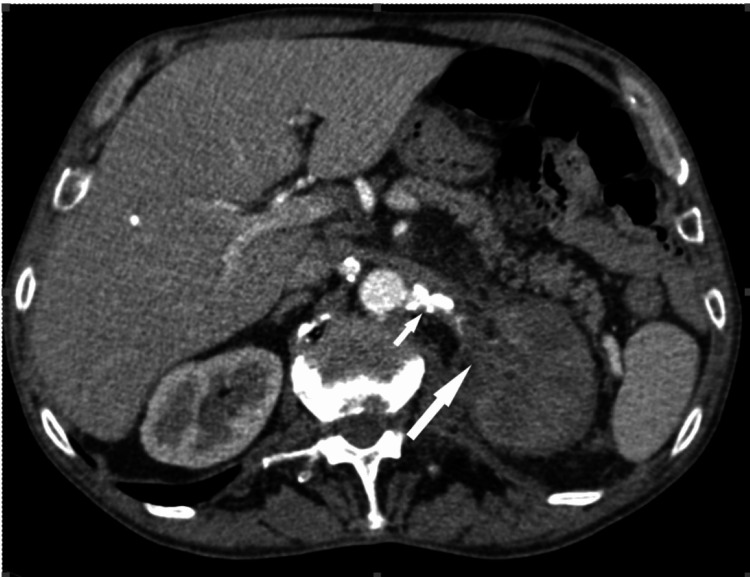
The contrast-enhanced CT (CECT) scan conducted during the arterial phase in the axial plane reveals a kidney devoid of distinguishable facial features, a characteristic manifestation attributed to transitional cell carcinoma (TCC) with infiltration into the sinus fat. Furthermore, notable within the imaging and indicated by the small arrow is the identification of renal artery stenosis, a condition induced by calcifications. This figure is the original work of the authors. Patient consent for the use of the image was obtained, as mentioned in the patient consent form (point no. 12).

During the initial phases of transitional cell carcinoma of the kidney (stage I or II), a solid mass forms in the center of the renal pelvis. This mass grows outward, putting pressure on the fat in the renal sinus and creating a visible separation from the renal parenchyma. This separation can be observed either through the presence of renal sinus fat or contrast material used in imaging. The disease gets worse in stages III and IV, though, and transitional cell carcinoma (TCC) spreads to the nearby renal parenchyma. This causes the renal sinus fat to disappear completely (Figure 2). Despite this, the natural form of the kidney is usually maintained [8].

Squamous cell carcinoma

Squamous cell carcinoma constitutes 1-7% of neoplasms in the upper urinary system. It is frequently associated with persistently infected staghorn calculi. Individuals afflicted with this syndrome typically exhibit advanced tumors that are poorly to moderately differentiated. The imaging characteristics of squamous cell carcinomas closely resemble those of transitional cell carcinomas. The presence of kidney stones in the past and chronic inflammation of the urothelium are significant factors that contribute to the development of squamous cell carcinoma. There may be squamous cell carcinoma present if there is a renal stone, a large renal sinus component, and an infiltrating lesion that looks like it is spread out geographically [9].

Adenocarcinoma

Adenocarcinoma comprises less than 1% of upper urinary tract malignancies. An adenocarcinoma may have a connection to calculi and long-term obstruction, since these symptoms may exist in patients with this type [10].

Mesenchymal tumors

**Figure 3:**
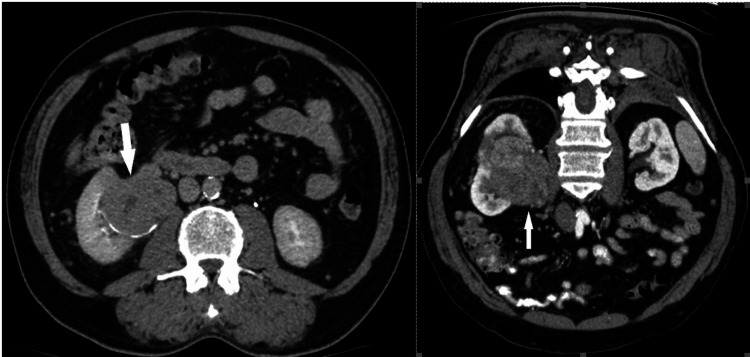
The axial contrast-enhanced CT (CECT) scan performed during the excretory phase and the coronal oblique scan conducted during the arterial phase both unveil the presence of a histologically confirmed leiomyosarcoma of the inferior vena cava (IVC). This pathology manifests as a substantial heterogeneous mass, predominantly owing to central necrosis, which exhibits diminished enhancement compared to the tumoral tissue. Additionally, the imaging reveals the invasion of the right renal sinus and right renal parenchyma by the sarcoma, resulting in a discernible mass effect on the collecting system. These observations collectively depict the intricate nature of the leiomyosarcoma, highlighting its propensity for invasion and its consequential impact on adjacent anatomical structures within the renal vicinity. This figure is the original work of the authors. Patient consent for the use of the image was obtained, as mentioned in the patient consent form (point no. 12).

Mesenchymal tumors are neoplasms that arise from pluripotent cells with the ability to differentiate into various types of mesenchymal tissues, including blood vessels, fibrous tissue, adipose tissue, and others. Adult renal mesenchymal tumors can originate in the renal sinus space, renal capsule, or renal parenchyma and can exhibit either benign or malignant characteristics. Angiomyolipoma, leiomyoma, and lipoma are instances of noncancerous mesenchymal tumors. Leiomyosarcoma (Figure 3) angiosarcoma, and malignant fibrous histiocytoma are types of malignant mesenchymal tumors [11].

Imaging studies can produce ambiguous outcomes when diagnosing these rare malignancies. Nevertheless, MRI possesses a clear superiority over CT scans in precisely depicting the extent of infiltration into the renal vein and inferior vena cava. CT scans may typically identify the presence of a mesenchymal tumor in the renal sinus. These images usually show a clear boundary between the tumor and the renal collecting system, as well as compression of the renal pelvis and calyces. Enhanced CT scans can also visualize constricted blood arteries in the renal hilum. Additionally, during the excretory phase of the scan, a compressed renal pelvis and calyces may be observed without any abnormalities in the filling process.

Parenchymal tumors

Most tumors that originate from the renal parenchyma grow by expanding and appearing as round masses. These tumors can also press against or infiltrate the renal sinus fat as they grow toward the renal pelvis. The common examples of these tumors are renal cell carcinoma (RCC) and benign multilocular cystic nephroma (MCN).

RCC

**Figure 4:**
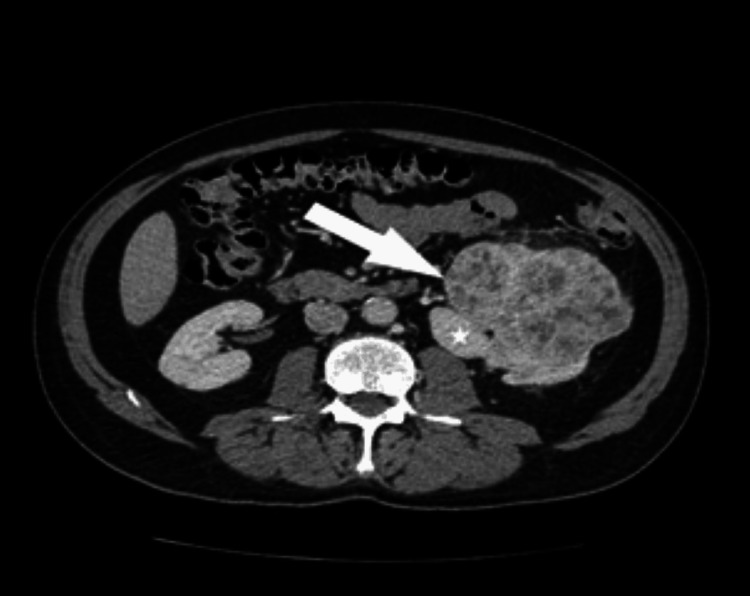
During the arterial (a) and urographic (b) phases of contrast-enhanced CT (CECT), a comprehensive evaluation unveils a considerable, expansive, and heterogenous soft-tissue lesion centrally situated within the left kidney. Notably, this lesion demonstrates invasion into the renal sinus. The heterogeneous appearance of the mass is attributable to variable enhancement patterns, with heightened enhancement evident in the periphery juxtaposed against lower enhancement centrally, which corresponds to areas of necrosis within the tumor. These characteristic features align closely with the diagnostic criteria for renal cell carcinoma (RCC). The left renal vein is marked by the asterisk at the renal sinus level. This figure is the original work of the authors. Patient consent for the use of the image was obtained, as mentioned in the patient consent form (point no. 12).

**Figure 5:**
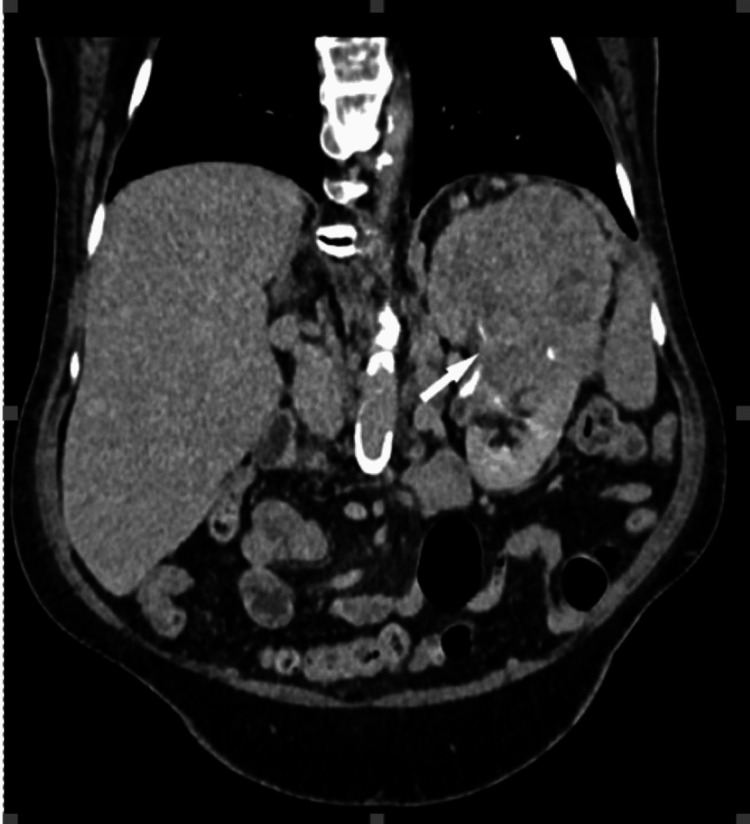
Coronal oblique urographic contrast-enhanced CT (CECT) phase presents a striking visual representation of a substantial mass originating from the upper two-thirds of the left kidney. This voluminous lesion exhibits characteristics indicative of necrosis, further emphasizing its significant size and advanced pathology. Notably, the mass is depicted invading the renal sinus. This figure is the original work of the authors. Patient consent for the use of the image was obtained, as mentioned in the patient consent form (point no. 12).

RCC is the most prevalent type of malignant renal tumor (Figure 4 and Figure 5); RCC is a primary malignant adenocarcinoma that originates from the renal tubular epithelium. On imaging tests, RCC can appear in different forms, from being solid and uniform to having areas of necrosis, cystic change, and hemorrhage.

Most cases of RCC grow by expansion and can often extend into the renal pelvis, causing focal hydronephrosis or displacement of the caliceal. In contrast to TCC, RCC has a tendency to spread into the venous system [12].

Three-dimensional CT or MR imaging can provide detailed information about the location of the renal mass, its relationship with the collecting system, and the renal vessels.

MCN

**Figure 6:**
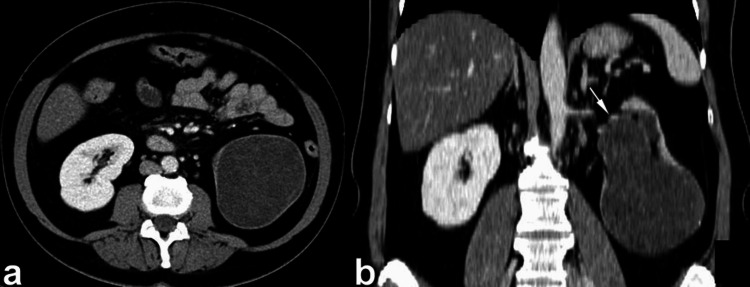
Axial picture (a) and the reconstruction in the frontal plane (b) of a contrast-enhanced CT (CECT) scan during the nephrographic phase unveil a notable pathological finding within the left kidney. Specifically, a multiloculated cystic mass is prominently displayed, indicating the presence of multiple septa. This mass is encapsulated by a robust fibrous capsule, suggestive of a well-defined boundary separating it from the surrounding renal parenchyma. Notably, the presence of this thick fibrous capsule exerts pressure on the adjacent renal parenchyma with cortical thinning. Furthermore, the capsule extends into the renal sinus, as indicated by the arrow, underscoring the invasive nature of the lesion. This figure is a republished image, permission was obtained from the original publisher, https://epos.myesr.org/poster/esr/ecr2018/C-2802

MCN (Figure 6) is generally characterized by a single cystic mass with multiple compartments, enclosed by a thick fibrous layer and compressing surrounding tissue. Such cysts do not often exhibit calcification, hemorrhage, or necrosis. On CT scans, MCN appears as a large, multiloculated cystic mass, often extending into the renal pelvis, with septal enhancement that may be inconsistent and without solid enhancements. Perirenal fat may or may not display streakiness [13]. Surgical treatment for MCN typically involves the removal of the affected kidney or a portion of it, along with any impacted lymph nodes.

Retroperitoneal tumors that extend to the renal sinus

The renal sinus is part of the perinephric space, so any retroperitoneal tumor can spread to the renal sinus. Several retroperitoneal tumors, including lymphoma, sarcomas, multiple myelomas, and Castleman disease, can directly invade the perinephric space and renal sinus.

**Figure 7:**
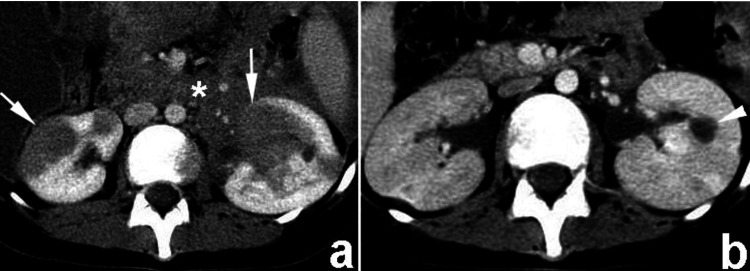
During the nephrographic phase, contrast-enhanced CT (CECT) imaging was conducted both before (a) and after (b) six months of treatment. The scans reveal the presence of a significant infiltrative mass of soft tissue, denoted by the asterisk, indicative of lymphoblastic lymphoma. According to its infiltrative pattern, the mass extends into the retroperitoneum and invades the renal sinus and parenchyma, as highlighted by the arrows. Following six months of treatment, a subsequent CT scan demonstrates complete resolution of both the renal and retroperitoneal tumors. Notably, attention is drawn to the identification of a small cyst located on the left kidney, denoted by the arrowhead. This figure is a republished image, permission was obtained from the original publisher, https://epos.myesr.org/poster/esr/ecr2018/C-2802

On CT scans, retroperitoneal lymphoma appears as an expansive tissue mass that encompasses the perinephric area and renal sinus. The renal vessels remain open despite being surrounded by the tumor, a feature that is typical of lymphoma, and obstructive hydronephrosis is often seen due to the direct involvement of the renal collecting system [14]. This appearance is most common in patients with advanced non-Hodgkin lymphoma (Figure 7).

Both on CT and ultrasonography, lymphomatous masses are uniform in appearance. CT scans show less enhancement of the mass, and on ultrasonography, lymphoma appears as a hypoechoic mass, reflecting its homogeneous tissue structure.

Metastasis

Metastasis to the sinus lymph nodes can occur either as a widespread retroperitoneal spread or as isolated involvement, as seen with primary gonadal tumors. This is due to the abundance of perforating vessels and lymphatic channels that connect to the renal sinus [15].

Lipomatosis

**Figure 8:**
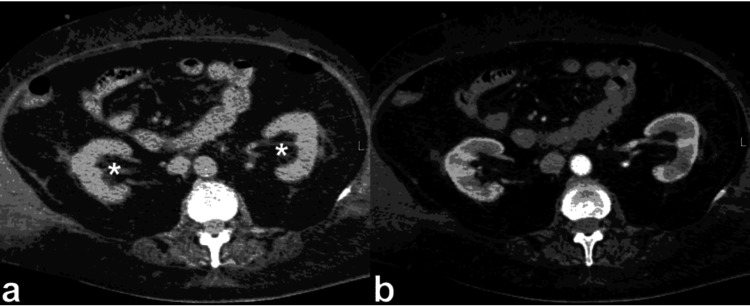
Non-enhanced CT (NECT) phase (a) and the contrast-enhanced CT (CECT) arterial phase (b) showcase the presence of extra pure fat replacement surrounding both sides of the renal sinus, depicted by the asterisk. However, despite the presence of this fat replacement, there is no discernible mass effect and no displacement observed on the urinary collecting system. This figure is a republished image, permission was obtained from the original publisher, https://epos.myesr.org/poster/esr/ecr2018/C-2802

The renal sinus usually has adipose tissue that surrounds multiple tissues inside it. Renal sinus lipomatosis (Figure 8) is defined as the pathological accumulation of adipose tissue in the renal sinus. The rise in fat content is a prevalent consequence of the aging process as well as a consequence of obesity. However, the rise in fat content can also be attributed to variables such as renal parenchymal shrinkage, inflammation, calculous illness, aging, or exposure to exogenous or endogenous hormones.

Certain individuals exhibit a more pronounced manifestation of massive renal sinus lipomatosis, characterized by the presence of infection, renal calculi, persistent hydronephrosis, and substantial shrinkage of the kidney's parenchyma [16].

Renal replacement lipomatosis is a rare disease in which fat cells grow in the renal sinus and perinephric area. This makes the renal parenchyma shrink and get badly damaged from the constant inflammation. Renal parenchymal atrophy or destruction, perinephric and hilar lipomatosis, renal calculi, and perinephric abscesses that may spread to the psoas muscle are among the characteristics of this illness that are typically shown on CT scans [17].

Renal cysts

**Figure 9:**
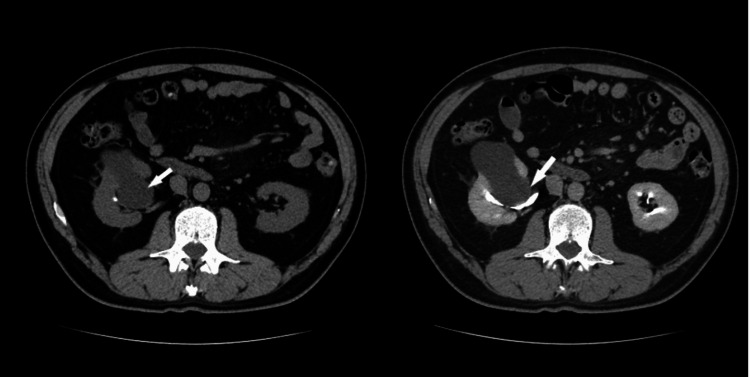
Non-enhanced CT scan (NECT) (a) and contrast-enhanced CT (CECT) scan during the urographic phase (b), a notable observation is evident. Specifically, there is the identification of a right parapelvic cyst, marked by the arrow, which protrudes into the renal sinus from the adjacent tissue. Despite its presence, there is no observed compression or pressure exerted upon the pelvicalyceal system. This figure is the original work of the authors. Patient consent for the use of the image was obtained, as mentioned in the patient consent form (point no. 12).

**Figure 10:**
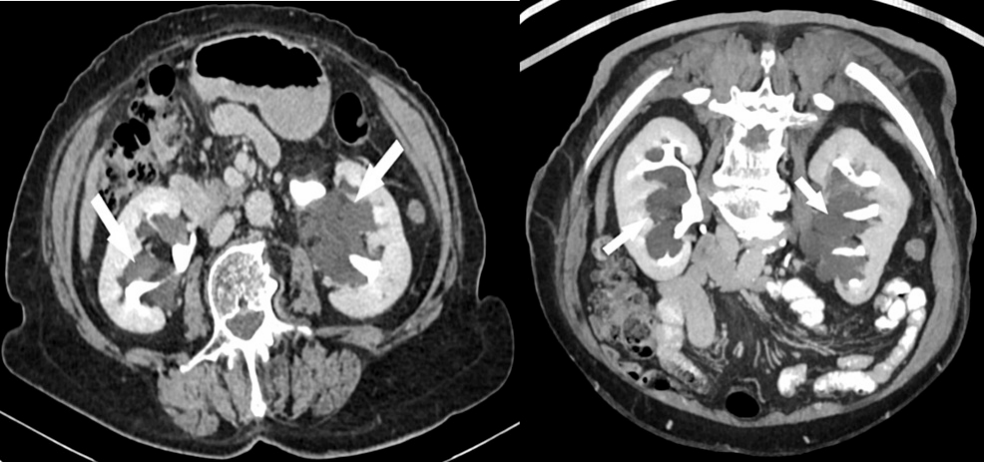
The axial and oblique coronal contrast-enhanced CT (CECT) images captured during the excretory phase reveal multiple cysts, bilaterally distributed and originating within the kidney parenchyma, which are prominently displayed. These parapelvic cysts extend into the renal sinus region, indicating their involvement with the anatomical structures surrounding the kidneys. This figure is the original work of the authors. Patient consent for the use of the image was obtained, as mentioned in the patient consent form (point no. 12).

**Figure 11:**
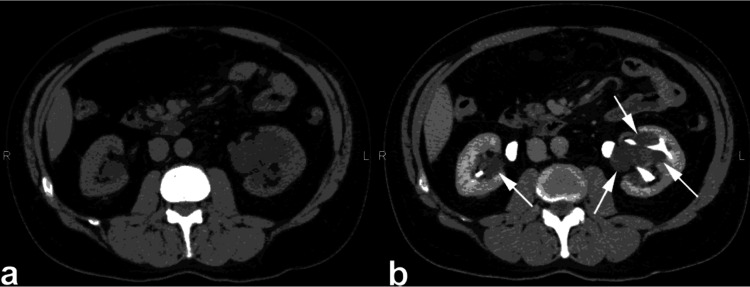
Non-enhanced CT (NECT) (a) and contrast-enhanced CT (CECT) during the urographic phase (b), showcase two peripelvic cysts, indicated by the arrows, which are evident on either side of the renal pelvis. These simple fluid-filled cysts are situated in close proximity to the renal pelvis and infundibula, with no observed compression or constriction of the pelvicalyceal system. This figure is the original work of the authors. Patient consent for the use of the image was obtained, as mentioned in the patient consent form (point no. 12).

Renal sinus cysts are a type of simple cyst that are located within the renal sinus and occur in just 1.28% to 1.5% of autopsy cases [18]. They can be classified into parapelvic (Figure 9 and Figure 10) and peripelvic cysts (Figure 11). Parapelvic cysts come from the adjacent renal tissue and project into the renal sinus, while peripelvic cysts originate within the sinus and have a lymphatic origin [18]. Sometimes, peripelvic cysts can put pressure on the pelvic floor and cause hydronephrosis. On the other hand, peripelvic cysts are usually bilateral and do not affect how the body works. They may be mistaken for hydronephrosis on non-contrast CT scans or ultrasonography but can be differentiated on contrast-enhanced CT scans [19].

Vascular pathologies

**Figure 12:**
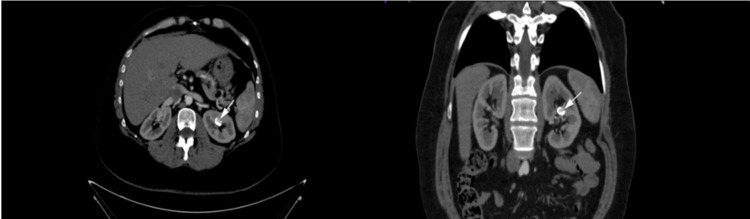
Contrast-enhanced CT (CECT) in the artery phase reveals significant findings in both axial (a) and oblique coronal-plane reconstructions (b). Notably, there is a renal artery saccular aneurysm, indicated by the arrows, which presents as a focal dilation and is engorged with contrast material, characteristic of a saccular aneurysm but with multiple parietal calcifications. This imaging modality provides a detailed visualization of the arterial vasculature, facilitating the precise identification and characterization of pathological conditions such as aneurysms. This figure is the original work of the authors. Patient consent for the use of the image was obtained, as mentioned in the patient consent form (point no. 12).

**Figure 13:**
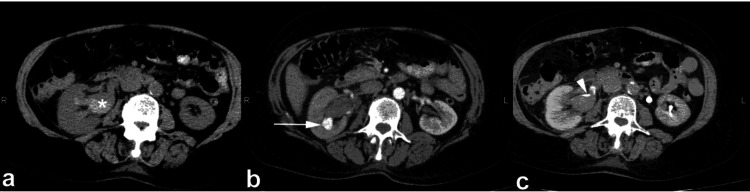
Non-enhanced CT (NECT) (a) is followed by a contrast-enhanced CT (CECT) scan of the artery (b), and subsequently, the urographic phase (c). In these sequences, an arrow highlights a contrast-filled outpouching detected within a branch of the right renal artery. Notably, blood is observed to be draining into the renal pelvis (asterisk), indicating the presence of a pseudoaneurysm with a fistula in the pyelocaliceal system. Additionally, attention should be paid to the iodinated contrast media that have been flushed out in the anterior portion of the renal pelvis (arrowhead), accentuating the anatomical details and aiding in the characterization of the vascular anomaly. This figure is a republished image, permission was obtained from the original publisher, https://epos.myesr.org/poster/esr/ecr2018/C-2802

Vascular problems, like renal artery aneurysms (Figure 12), vein-artery connections, renal vein varix, and arteriocaliceal fistula (Figure 13), can affect the renal sinus and show up as pseudocystic lesions near the pelvis. The utilization of contrast-enhanced CT or MRI, color Doppler ultrasound, and angiography can readily detect these conditions.

**Figure 14:**
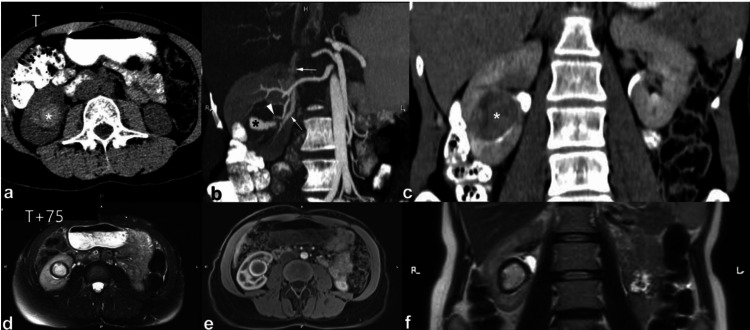
A comprehensive imaging assessment was conducted using multiple modalities, including non-enhanced CT (NECT) (a), contrast-enhanced CT (CECT) in the arterial phase with reconstruction in an oblique plane (b), CECT in the urographic phase with reconstruction in the frontal plane (c), MRI axial T2 half-Fourier acquisition single-shot turbo spin echo imaging fat suppression (HASTE FS) (d), MRI axial Volumetric interpolated breath-hold examination (VIBE) post-gadolinium in the arterial phase (e), and MRI coronal T2 half-Fourier acquisition single-shot turbo spin echo imaging (HASTE) (f). In the case of a 35-year-old woman who recently gave birth, abnormalities in renal arterio-venous communication with associated pseudoaneurysms were identified, denoted by black asterisks. Specifically, the feeding artery (arrowhead) and venous drainage (arrows) exhibited notable anomalies, indicative of the presence of pseudoaneurysms. Additionally, a lesion resembling a hematoma in the kidney region was observed, characterized by the presence of spontaneous hyperdensities compatible with acute hemorrhage. Following a 75-day interval, subsequent MRI imaging revealed a significant resolution of the malformation, indicating spontaneous regression. Moreover, the hematoma appeared to have diminished in size. This figure is a republished image, permission was obtained from the original publisher, https://epos.myesr.org/poster/esr/ecr2018/C-2802

There are three types of arteriovenous fistulas in the kidneys: congenital arteriovenous fistulas, which are there at birth; postpartum acquired arteriovenous fistulas (Figure 14), which form after a procedure like a biopsy or an injury; and idiopathic arteriovenous fistulas, whose cause is unknown [20]. The majority of these connections are acquired, with biopsies being the most frequent cause. While aortic-venous connections in the kidneys generally remain asymptomatic, certain individuals may manifest symptoms including but not limited to heavy hemorrhage, abdominal whooshing, heart failure, hypertension, or abdominal discomfort. Depending on the dimensions of these connections, imaging tests may fail to detect them at times [21].

Hematomas

**Figure 15:**
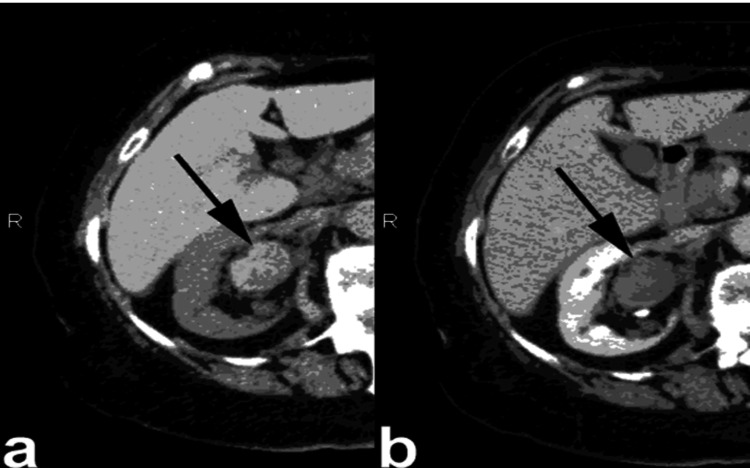
Imaging studies comprising non-enhanced CT (NECT) (a) and contrast-enhanced CT (CECT) during the urographic phase (b). These modalities were utilized to evaluate a rare complication associated with blood thinners: a right kidney sinus hematoma. The NECT scan (a) provided an initial assessment, revealing the presence of a hematoma within the right kidney sinus. Subsequent CECT during the urographic phase (b) further delineated the extent and characteristics of the hematoma, aiding in its precise characterization and assessment of potential complications. This figure is a republished image, permission was obtained from the original publisher, https://epos.myesr.org/poster/esr/ecr2018/C-2802

Hemorrhage can result from trauma, arteriovenous malformation, or anticoagulant medication. The formation of a renal sinus hematoma, which is characterized by bleeding within the renal sinus, is an unusual side effect of anticoagulant therapy (Figure 15). This problem may emerge in those who have a long prothrombin time. CT scans show the presence of an irregular, thick substance that clearly supports the presence of recent blood and does not amplify when contrast is used [22].

Urinomas

Urinomas are collections of urine commonly located in the perirenal space in the retroperitoneum. They are usually caused by blockage of the urinary tract, trauma, or after medical instruments are used. In contrast-enhanced studies during the excretory phase, urine leakage can usually be directly seen due to the contrast material extravasating from the urinary tract [23].

Urolithiasis

**Figure 16:**
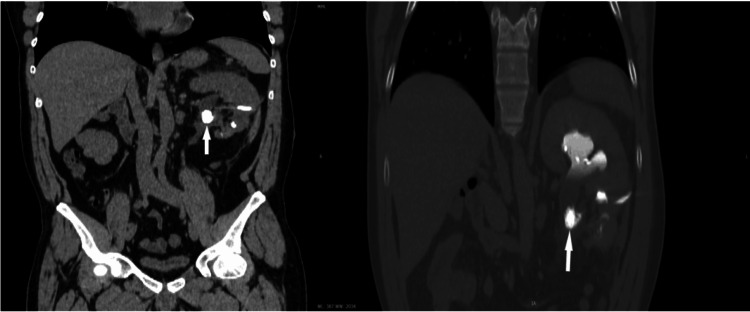
Non-enhanced CT (NECT) (a) and contrast-enhanced CT (CECT) during the urographic phase (b). The NECT scan (a) indicates the presence of a calculus within the left renal pelvis (which also happens to show a duplex collecting system, both pathways being obstructed by the calculus), clearly demarcated by the arrow. Subsequent imaging with CECT during the urographic phase (b) showcases the dilatation of the pelvicalyceal system (hydronephrosis). There are also visible partial fragments of nephrostomy tubes which should not be mistaken for calculi. This figure is the original work of the authors. Patient consent for the use of the image was obtained, as mentioned in the patient consent form (point no. 12).

**Figure 17:**
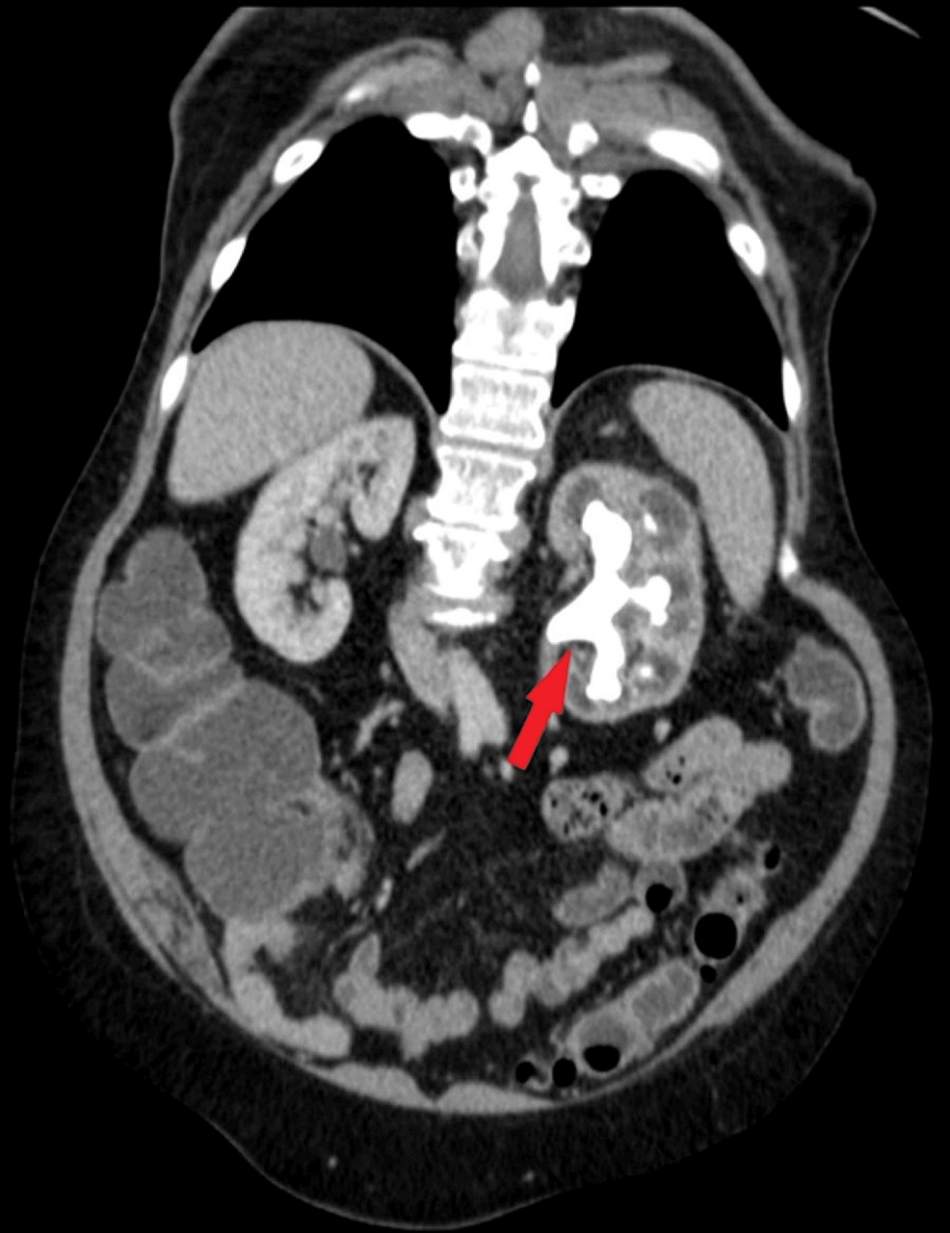
Coronal oblique contrast-enhanced CT (CECT) during the nephrographic phase illustrates a complete staghorn calculus, which is prominently tapering the upper collecting system. This figure is the original work of the authors. Patient consent for the use of the image was obtained, as mentioned in the patient consent form (point no. 12).

A common cause of calcifications in the kidney is kidney stones (Figure 16 and Figure 17). One in 10 people is affected at least once during their lifetime [24]. Calcium oxalate is the main component of kidney stones, however demographic and metabolic variables might affect it. A non-contrast CT scan can identify most kidney stones because they are radio-dense despite fluctuations in their density. Uric acid stones have a density of 100-200 HU, while calcium oxalate stones have 400-600 HU.

Inflammation and inflammatory pathologies

In severe or chronic pyelonephritis, renal inflammatory diseases often spread to the renal sinus and surrounding territory. Primary inflammatory lesions are rare. Renal sinus inflammation can resemble a tumor [25].

**Figure 18:**
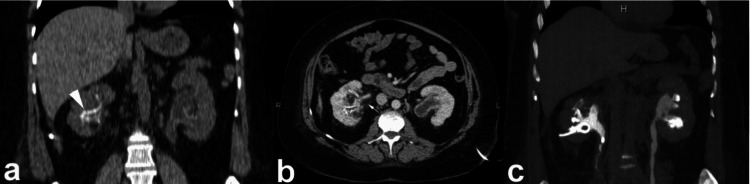
Non-enhanced CT (NECT) scan (a), a contrast-enhanced CT (CECT) scan in the nephrographic phase (b), and a urographic phase (c). The NECT scan (a) reveals wall calcifications within the pelvicalyceal systems and proximal ureters, as denoted by the arrowheads. Subsequent imaging with CECT during the nephrographic phase (b) provides enhanced visualization of the inflammatory changes within the pelvicalyceal systems and proximal ureters. Collectively, these imaging findings support the diagnosis of encrusted uretero-pyelitis. There are also multiple calculi showcased during the excretory phase CECT. This figure is a republished image, permission was obtained from the original publisher, https://epos.myesr.org/poster/esr/ecr2018/C-2802

Due to recurrent urinary tract infections, usually caused by Corynebacterium urealyticum, calcifications in the pelvicalyceal system and ureter create encrusted uretero-pyelitis (Figure 18), an uncommon and severe condition. Calcium deposits on CT images aid diagnosis.

Miscellaneous pathologies

Metal Toxicity

**Figure 19:**
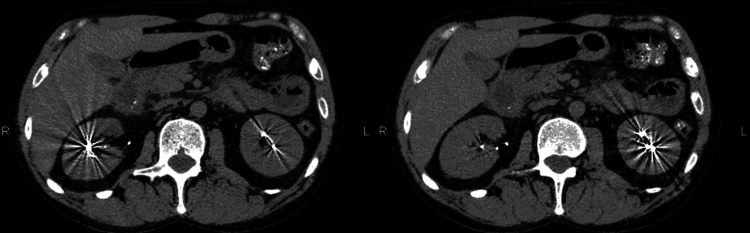
Non-enhanced CT (NECT) scans in the axial plane (a and b) depict a person who has been poisoned by heavy metals, exhibiting characteristic metallic artifacts on both sides. Heavy metals such as lead, mercury, arsenic, and cadmium can accumulate within the body tissues, including the bones and organs, leading to toxic effects. The metallic artifacts produce characteristic radiopaque shadows. This figure is a republished image, permission was obtained from the original publisher, https://epos.myesr.org/poster/esr/ecr2018/C-2802

The detection of metal objects in an imaging scan can cause severe, streak-like distortions (Figure 19). These occur because the metal has a density that exceeds the computer's capacity to handle, resulting in an inadequate reduction of image intensity.

## Conclusions

The renal sinus can have many pathological diseases, thus excretory urography, ultrasound, CT, MRI, and angiography are needed to diagnose it quickly. Out of all the modalities, CT imaging with an IV contrast medium is essential for diagnosing and assessing renal sinus diseases. CT imaging helps characterize lesions and guide patient care by revealing their density and extent. With the help of CT imaging, we can characterize correctly the etiology of the plethora of pathologies that involve the renal sinus. Effective treatment requires knowledge of renal sinus pathology imaging and differential diagnosis. 

## Figures and Tables

**Figure 20:**
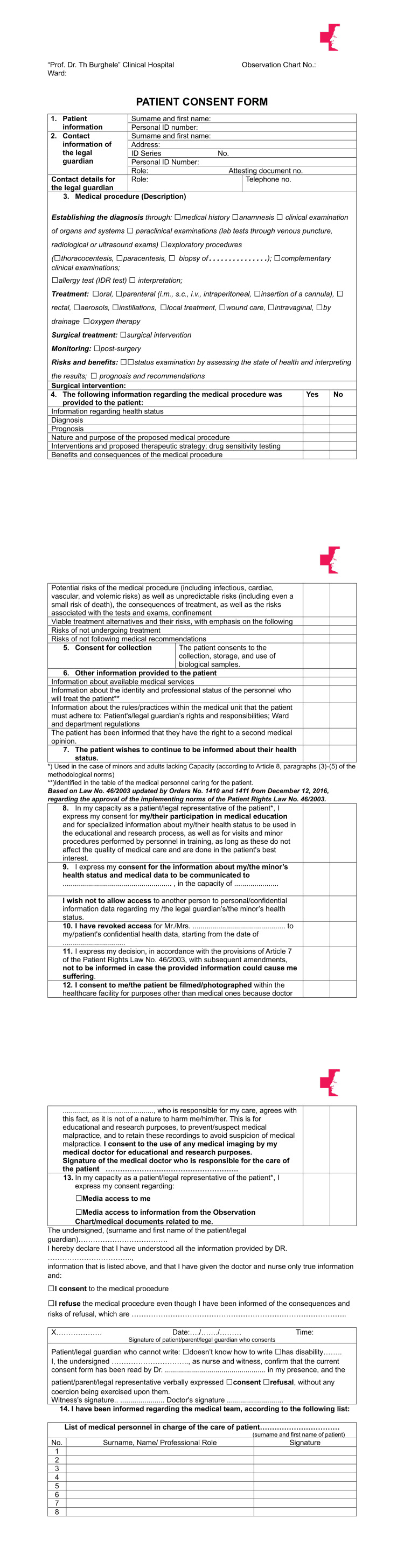
Patient consent form

## Appendices

Consents from patients for the use of the images were obtained, as mentioned in point no. 12 of the patient consent form (Figure 20).
